# Associations of Medical Visits with Dentist Visits: A Register-Linkage Study of a Working-Age Population in Finland

**DOI:** 10.3390/ijerph182413337

**Published:** 2021-12-18

**Authors:** Mikko Nurminen, Jenni Blomgren

**Affiliations:** Research Unit, The Social Insurance Institution of Finland, P.O. Box 450, 00056 Helsinki, Finland; jenni.blomgren@kela.fi

**Keywords:** medical visits, dentist visits, dental attendance, joint utilization, medical and dental service

## Abstract

Studies have usually addressed the utilization of either medical or dental services, and less is known about how medical and dentist visits are associated. As oral health is linked to systemic health, knowledge on care coordination between dental and medical services is important to gain understanding of the overall functioning of health care. Register data on 25–64-year-old residents of the city of Oulu, Finland, were used for the years 2017–2018 (N = 91,060). Logit models were estimated to analyze the probability of dentist visits, according to the number of medical visits in total and by three separate health care sectors. The majority, 61%, had visited both a medical professional and a dentist. All sectors combined, as few as one to two visits increased the odds of dentist visits (OR: 1.43, CI: 1.33, 1.53). When separated by medical professionals’ health care sectors, for one to two visits, the strongest association was found with public (OR: 1.17, CI: 1.12, 1.22) and private sector (OR: 1.35, CI: 1.30, 1.41). For occupational health service visits, the odds increased only after six or more visits. The results support the idea of integrated medical and dental care. However, the result may also arise from individual health behavior where health-conscious persons seek both medical and dental care independently.

## 1. Introduction

Utilization of health care has been shown to have a strong social gradient. Irrespective of need of care, those with higher socioeconomic status are more likely to utilize medical and dental services [1,2,3,4,5]. At the same time, among those who use health care, the most frequent health care attenders have been shown to come from vulnerable groups in terms of chronic sickness [6,7] and socioeconomic background [7,8,9]. While the focus has been on the socioeconomic, morbidity, and demographic characteristics among attenders of either medical or dental care [1,2,3,4,5], very little is known about how the utilization of medical services itself is related to the attendance of dental health care. The links between oral health and general health and well-being [10,11,12] indicate the salience of coordinated care between physicians and dentists. Thus, for the overall assessment of the functioning of the health care system, it is necessary to gain knowledge on how utilization of medical services predicts the utilization of dental services.

In most countries, health care is provided separately through public and private sector schemes. Studies have shown that persons from a higher socioeconomic background utilize disproportionately more private sector care [4,5,13,14], while those from a poorer socioeconomic background utilize more public sector care [2,4,5,13,15]. These differences may partly explain the polarization of health service utilization by socioeconomic background [16,17]. Further polarization may be introduced if medical services in one sector also more strongly promote dental attendance (or vice versa). In terms of efficient and equitable allocation of health care resources, comprehensive information is needed on whether different medical sectors promote dental care utilization in different ways.

The setting that we studied in this paper, the Finnish health care system, is unique in the sense that it is organized in three co-existing sectors: a public sector, occupational health services (OHS), and a private sector [18]. Medical services are provided in all three sectors, while dental care is typically only provided in the public and private sectors. In general, the utilization of one sector does not preclude the utilization of the other sectors.

Public health care consists of universal coverage for all Finnish residents. Public primary care is organized by municipal health centers, while specialized health care is organized by larger hospital districts. Specialized health care may be accessed through primary care, with nurses and physicians acting as gatekeepers. The funding is based on taxes and small co-payments for the treatment [18].

OHS provides health care services for the working population. In Finland, all employers are obliged to arrange at least preventive care for their employees. However, the range of care covered in OHS can vary between employers, and primary care level coverage is commonly included [19]. OHS is mainly arranged through private companies but can be also arranged directly by the employer or purchased from a public health center. The expenses are covered by employers and statutory tax-like health insurance payments, collected jointly from employees and employers.

The private health care sector is a market-based fee-for-service system, although part of the fees is also covered by the National Health Insurance (NHI) scheme. In 2018, the average fees per visit for physicians and dentists were 106 and 155 euros, respectively [20]. For these visits, the average amounts covered by the NHI scheme were 16 and 22 euros, respectively [20]. The NHI reimbursement rates have decreased over the years, and since 2016, expenses for dental examinations are covered only every other calendar year. Waiting times in the private sector are typically shorter than in the public sector and specialists can be accessed without gatekeeping.

Previous studies have focused on either the sectoral utilization of medical services [1,2,4] and frequent attenders [6,7,8,9], or the sectoral utilization of dental care [5,13,14,15]. Studies that have addressed the joint-utilization of medical and dental services are scarce and survey-based [21,22,23,24], and have not taken into account the different health care sectors. Comprehensive health care requires both services as oral health may crucially affect general health and the quality of life. More coordinated and integrated care has the potential to increase access and attendance in health care and oral care services, improve the efficiency of the services and, consequently, increase overall population health. However, the divide between the practice of medicine and dentistry has been historically prominent [23], and, thus, more up-to-date information on this subject is needed. To narrow the gap in this knowledge, the aim of this study was twofold: first, to study how utilization of medical services is associated with utilization of dental services; and second, whether there are differences in the association between sectors. Using register-based data, we analyzed how the total number of medical visits predicted the probability of dentist visits, and whether there were notable differences between the three medical sectors in predictive power.

## 2. Materials and Methods

### 2.1. Study Population

Our study population consisted of working age residents in the city of Oulu, Finland, during the 2017–2018 period. Oulu is the fifth largest city in Finland, with approximately 200,000 residents in 2018. Oulu does not differ from the general Finnish population in any systematic way in terms of demographic, socioeconomic, or health care utilization related factors [25]. The population data was gathered from the registers of the Social Insurance Institution of Finland. In the study population, we included individuals who were aged 25–64 at the beginning of 2017. We also excluded those individuals who were students at the beginning of 2017 according to the information gathered from the registers of Statistics Finland. In Finland, students form their own small health care subsystem from which we had no data. The final sample consisted of 91,060 residents.

### 2.2. Data on Outpatient Medical Visits and Dental Care Visits

Data on outpatient medical and dental care visits were gathered from several different registers for the 2017–2018 period. We chose a two-year period since it is common to have regular dental checks only every other year. Using data from two consecutive years allows us to examine more closely those who rarely utilize health care. Also, private dental care is reimbursed by the NHI scheme only every other year.

First, data on public outpatient medical care and dental care utilization were obtained from the register of the city of Oulu. For outpatient medical care, we restricted the data to care given by physicians and nurses. For brevity, we use the term “medical” to cover the care given by both physicians and nurses. For dental care, we restricted the data to care given by dentists. This register included visits with appointment and on-call duty visits. In addition, we included information on visits with appointment and on-call duty visits to outpatient specialized health care. That information was derived from the Care Register of Health Care, which is maintained by the Finnish Institute for Health and Welfare.

Second, we gathered data on OHS utilization from the four largest OHS providers in Oulu (Attendo, Mehiläinen, Terveystalo, and Työterveys Virta). Together they cover around 92% of all OHS visits in Oulu [26].

Third, the information on private sector medical and dental care utilization was derived from the registers of the Social Insurance Institution of Finland. These data included all visits and procedures that were reimbursed by the NHI scheme.

We included only visits that were active face-to-face visits (contacts by phone, for example, were not included). In the registers, one visit may include several inconsistently recorded events, and it was not possible to measure reliably the number of separate visits during one day with the same service provider. Thus, we approximated the number of visits as the number of separate contact days with each health care provider during the two-year period.

### 2.3. Covariates

Table 1 lists the other covariates used in this study. Information on sex, age, education, and occupational class was retrieved from Statistics Finland. We divided education into four different levels: upper tertiary, lower tertiary, secondary, and basic. We divided occupational class into five different categories according to Statistics Finland [27]: upper non-manual employee (e.g., directors, physicians, and teachers), lower non-manual employee (e.g., nurses and technicians), manual worker (e.g., construction workers and mechanics), entrepreneur, and other. This final group, other, included unemployed and retired persons as well as those with missing information.

Information on personal gross taxable income, added together for the two years from 2017 to 2018, was collected from the Finnish Tax Administration. To take into account any nonlinearities, we grouped income into five categories, from highest to lowest, by quintiles.

As a proxy for chronic morbidities, we used the number of entitlements to special reimbursement for medicine expenses. This information was gathered from the register of the Social Insurance Institution of Finland. To become entitled for the special reimbursement, a patient needs to apply for it and has to have a medical certificate from a physician. Once an affirmative decision has been given, a patient is entitled to a higher NHI reimbursement rate for medicine expenses. Information on these entitlements is often used as a register-based measure of chronic disease [28].

### 2.4. Statistical Methods

In this study, we were interested in the overall probability of dentist visits. Thus, we grouped visits to public and private dentists in order to form a binary variable of having any dentist visits during the two-year period. For medical visits, we grouped the number of visits during the two-year period into five categories: 0, 1–2, 3–5, 6–10, and over 10 visits. We calculated the total number of visits and their number by health care sector. This enabled us to study whether, with respect to dentist visits, there were any differences between health care sectors or nonlinearities in the number of medical visits.

First, we calculated the proportion of residents that had visited a dentist, a medical professional, or both during the two-year period from 2017 to 2018. Further, we calculated the proportion of residents that had a dentist visit with respect to the number of medical visits in each sector.

Second, to adjust the probability of having dentist visits for socioeconomic, demographic, and health related factors, we estimated a logit model by maximum likelihood [29]. The covariates included in the regressions were, in addition to the grouped medical visits, sex, age group, educational attainment, occupational class, income quintile, and the number of entitlements to medicine special reimbursement. These factors have been shown to be associated with dentist and medical visits [1,2,3,4,5], and if left uncontrolled, can introduce bias to the estimated medical visit parameters. The results were shown as odds ratios with their 95% confidence intervals. Due to the count data nature of dentist visits, we additionally estimated a negative binomial regression model. The results for medical visits from the negative binomial model were similar to the logit. The results are shown in the additional file Table S1. All analyses were conducted using R programming language version 4.1.0 [30].

## 3. Results

### 3.1. Distributions of Medical and Dentist Visits

Table 2 shows the descriptive statistics for medical and dentist visits. The mean total number of visits during the two-year period from 2017 to 2018 was 13.8 for medical visits and 2.2 for dentist visits. Visits to public and OHS medical professionals were more common than visits to private medical professionals. Less than 10% had no medical visits. A majority (65%) of the residents had at least one dentist visit. Over half of the residents (61%) had visited both a medical professional and a dentist. Only 5% had neither medical nor dentist visits.

**Figure 1:**
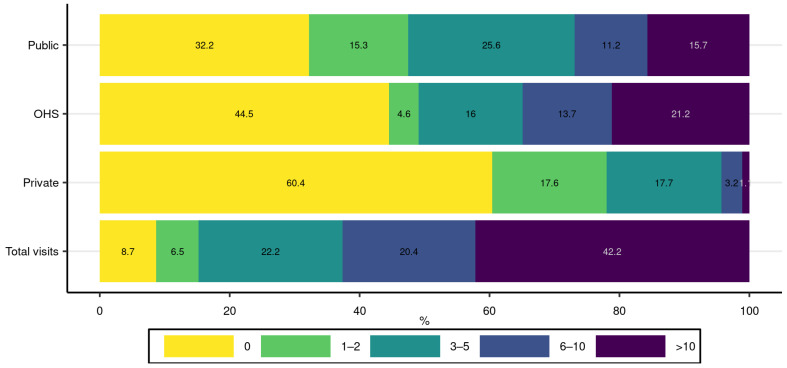
Proportion of residents with respect to number of medical visits in different sectors. Note that a resident may have medical visits in more than one sector.

To further describe the distribution of medical visits in different health care sectors during the two-year period, Figure 1 displays the proportions of residents with respect to the number of medical visits in each sector. It was common to have at least six or more medical visits in the public and OHS schemes while only a small proportion of persons had a large number of private medical visits. Approximately 22% had at least three visits to the private sector. All schemes combined, 42% had more than ten visits during the two-year period.

**Figure 2:**
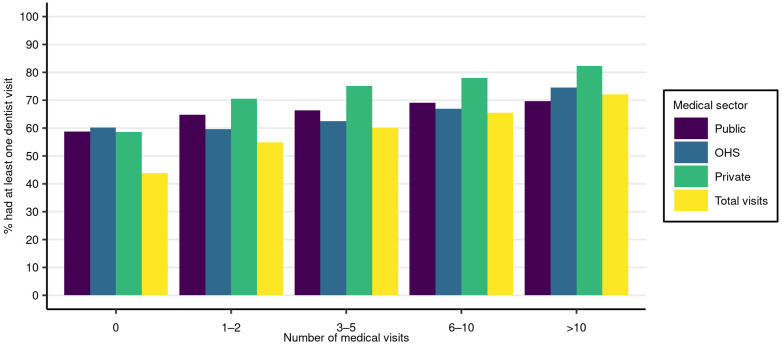
Proportions of residents with a dentist visit, by number of medical visits and sector. The proportions for each bar are calculated separately within each medical visit bin and sector. Note that a resident may have medical visits in more than one sector.

Figure 2 shows the proportion of residents with at least one dentist visit with respect to the number of medical visits, divided by sectors. The higher the number of medical visits, the more likely it was also to have a dentist visit during the examined two-year period. Of residents who had, in total, more than ten medical visits, 70% also had at least one dentist visit. Among those who had no medical visits, slightly over 40% had a dentist visit. However, as shown in Figure 1 above, the proportion of residents with no medical visits was small. The probability of dentist visits increased clearly with an increasing number of private medical visits, while the increase in the number of public or OHS medical visits was more moderate.

### 3.2. Adjusted Associations of Medical and Dentist Visits

**Figure 3:**
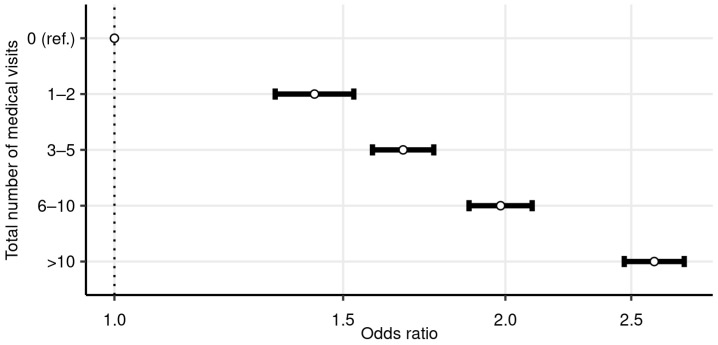
Logit results for dentist visits, by total number of medical visits (odds ratios with their 95% confidence intervals). Dependent variable is a binary variable indicating whether the resident has at least one dentist visit. Covariates included in the regression but not shown in the figure are sex, age group, education, occupational class, income quintile, and the number of entitlements to special medicine reimbursement. The *x*-axis is log-scaled. The sample size is 91,060.

Figure 3 presents the covariate-adjusted odds ratios from the logit model, with the probability of dentist visits as the dependent variable. Table A1 Model 1 shows the full table of results, including parameter estimates from the covariates not shown in Figure 3. Supplementary file Table S1 shows the results from a corresponding negative binomial model. The parameters of interest were the grouped total number of medical visits. Similar to the unadjusted results for the proportions above, the odds ratio increased with the number of visits. Increasing the number of medical visits from zero to one to two visits introduced a clear jump in the odds; after that the increase in the odds was steadier. Compared to those with no visits, the odds ratio was 1.43 (95% CI: 1.33, 1.53) for those with one to two visits, and 2.60 (95% CI: 2.47, 2.75) for those with over 10 visits.

**Figure 4:**
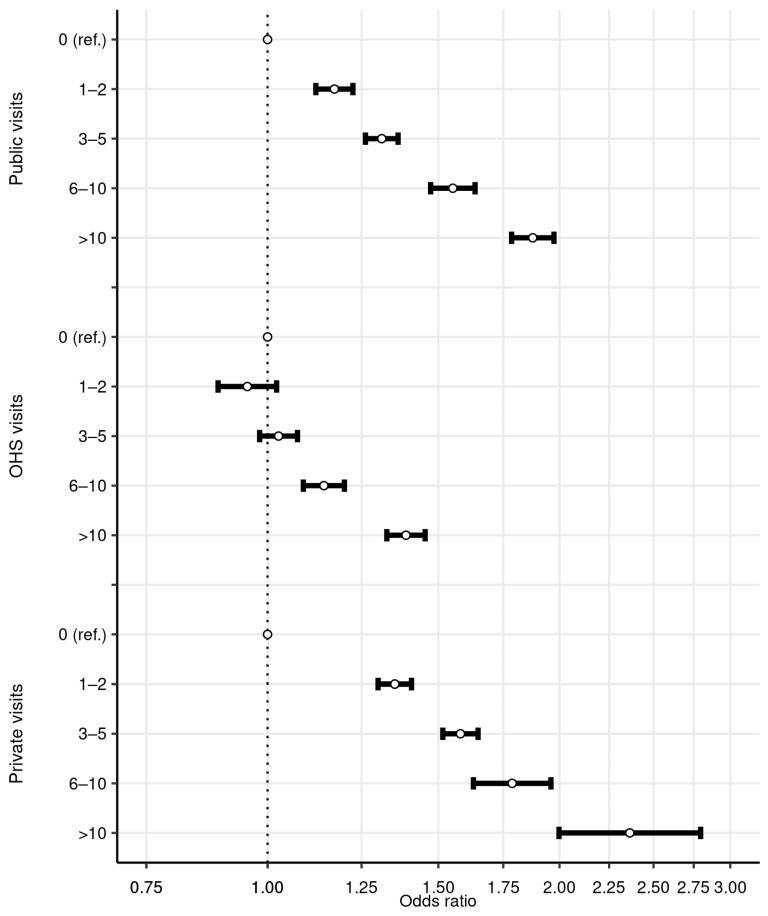
Logit results for dentist visits, by number of medical visits in separate sectors (odds ratios with their 95% confidence intervals). Dependent variable is a binary variable indicating whether the resident has at least one dentist visit. Covariates included in the regression but not shown in the figure are sex, age group, education, occupational class, income quintile, and the number of entitlements to special medicine reimbursement. All parameters are estimated from the same regression. The *x*-axis is log-scaled. The sample size is 91,060.

Figure 4 shows the results from a similar logit model but now with the number of physician visits separated to their respective sectors. Table A1 Model 2 shows the full table of results, including parameter estimates from covariates not shown in Figure 4. Supplementary file Table S1 shows the results from a corresponding negative binomial model. For all three sectors, a higher number of visits was associated with higher odds of a dentist visit. The increase was most prominent in public and private sector physician visits. For the OHS scheme, the increase was not significant until after at least six visits. The odds ratio was 1.31 (95% CI: 1.26, 1.36), 1.03 (95% CI: 0.98, 1.07), and 1.58 (95% CI: 1.52, 1.65), respectively, for three to five public, OHS, and private visits. While the increase in odds was largest for private physician visits, it was also more unusual to have a large number of private sector visits (Figure 1), and most residents only had zero to two visits in this sector.

## 4. Discussion

This study examined the association between medical visits and dental attendance during a two-year period from 2017 to 2018, using register data from the city of Oulu, the fifth largest city in Finland. The strength of the association was evaluated separately for public, OHS, and private medical visits. Crude descriptive methods and logistic regressions were used in the analysis.

The majority of residents, 91%, had at least one medical visit and most of the visits were made to the public or OHS sector. Almost two thirds had at least one dentist visit during the examined two-year period. Only 5% had neither medical nor dentist visits, and 61% had both medical and dentist visits. The fraction of patients that had a dentist visit consistently increased with the number of medical visits. While the increase was more pronounced with respect to private sector medical visits, one must bear in mind that the proportion of residents with a high number of private visits was notably lower compared to other sectors. The results of our logistic regression analysis revealed a similar result. The odds of a dentist visit increased with the number of medical visits. The association was strongest for private medical visits and lowest for OHS medical visits.

The association of medical visits in different sectors with dental care attendance has not been studied before. However, the general result that medical service utilization is associated with increased probability of dental attendance is in line with the scarce literature [22,23,24]. One possible reason behind the association of a higher number of medical visits with a higher likelihood of dentist visits could be that medical visits may promote health knowledge and better oral health [23,24]. A physician may urge the patient to visit a dentist. Furthermore, higher utilization of medical services may be related to greater overall need of health services or a more positive attitude towards health services [24]. Non-attendance in both services could be related to socioeconomic status. The lower the socioeconomic status, the lower is the probability of not using medical services [4] and dental services [5].

In contrast, as few as one to two medical visits were enough to cause a clear increase in the odds of dental attendance compared to those with no medical visits. This may be indicative of the same phenomena we discussed above; those having at least some medical visits are overall more inclined to look after their health. However, persons with only a few medical visits are likely to be relatively healthier and their dentist visits may often be related to routine checkups and preventive measures. Conversely, as the number of medical visits increases, the person is likely to be less healthy and the treatment of the underlying disease may include both physician and dental care. Those who had a large number of medical visits may be more likely to suffer from chronic conditions such as diabetes. In these types of cases, it is likely that treatment requires the regular involvement of both physicians and dentists [31,32,33].

It is also possible that the association between medical and dentist visits is stronger within sectors than across sectors (i.e., visits to public (private) medical professionals lead to public (private) dentist visits). This could partly explain the weaker association between OHS medical visits and the odds of having a dentist visit, as it is atypical for the OHS scheme to cover dental care. Further studies are needed to examine within-sector health care utilization. Another reason could be that the ease of access and free of charge visits lower the barriers to visit OHS physicians for less serious conditions. Also, employers may often require proof of sickness from their employees even for common minor diseases, such as flus, which then increase the number of OHS medical visits. These types of visits are unlikely to be strongly associated with dental care attendance.

Furthermore, lower socioeconomic status has been associated with poorer health [34,35,36,37,38] and utilization of public sector services. Thus, patients with chronic conditions and multiple diseases who require both medical and dental care may often be treated in the public sector. This could partly explain the strong association between increased public medical visits and dental attendance. In contrast, those who utilize more private sector services are likely to have a higher socioeconomic status [4,5,13,14]. They may also be more health conscious, which in turn may contribute to an increased probability of regular dental checks. 

The strength of this study is the detailed register data gathered and merged from several different administrative registers that offer a comprehensive view of both medical and dental visits in all of the main health care schemes in Finland. The advantage of using register-based data is the reliability in terms of negligible missing information and no selection into the data. By contrast, survey-based data may often suffer from response bias. Loss of data from dentist and medical visits and incomplete recall of past visits can be a major problem in studies that rely on survey data. In this study, we credibly observed visits to both medical care and dental care that reveal the real magnitudes of visits across the heath care sectors. With the demographic, socioeconomic, and morbidity information drawn from the registers, we were able to adjust for the common confounders known to be associated with health care utilization [1,2,3,4,5]. However, due to a lack of an experimental design, our estimated results did not have a causal interpretation. Even with the rich set of controls in the regressions, unobserved factors that are simultaneously correlated with both medical and dentist visits can bias our results. Also, due to this, it is not clear which way the direction of causality runs (i.e., from medical professionals to dentists or from dentists to medical professionals). It is likely to be very much case dependent, and in some cases, it could be that dentist visits lead to medical visits rather than the other way around.

One drawback in the data was the lack of information on the reasons behind the visits and the diagnoses associated with the visits. Without such information, little can be inferred about the health of the patient or the intensity of the treatment given. Thus, it could not be determined to what extent decisions were driven by a person’s own health behavior as opposed to underlying diseases. More research on the association between health and joint-utilization of medical and dental services is needed.

## 5. Conclusions

This study produced new information on how medical visits in different health care sectors are associated with the probability of dental attendance. Research on this topic is scarce as the literature has focused on studying the sociodemographic determinants of dental attendance. The more medical visits a person had during the two-year study period, the higher was the probability to have visited a dentist. Particularly, a high number of visits in the private and public sectors was strongly associated with dental attendance.

Our results suggest that medical visits may actively promote dental attendance. Also, our results could partly be explained by personal health behavior or by a greater overall need of comprehensive medical intervention that requires both medical and dental services. Future work needs to be undertaken to disentangle the importance of these factors in dental care utilization.

## Figures and Tables

**Table 1:** Descriptive statistics for the covariates of working-age (25–64) residents of Oulu in 2017–2018.

	N	%	Mean	Median
Sex				
Male	46,550	51.1		
Female	44,510	48.9		
Age group				
25–34	23,391	25.7	29.7	30.0
35–44	24,113	26.5	39.3	39.0
45–54	22,114	24.3	49.6	50.0
55–64	21,442	23.5	59.4	59.0
Education				
Upper tertiary	17,391	19.1		
Lower tertiary	26,206	28.8		
Secondary	38,184	41.9		
Basic	9279	10.2		
Occupational class				
Upper non-manual employee	21,560	23.7		
Lower non-manual employee	25,006	27.5		
Manual worker	15,139	16.6		
Entrepreneur	5531	6.1		
Other	23,824	26.2		
Income quintile				
Income quintile 5	18,212	20.0	161,352.1	131,323.4
Income quintile 4	18,212	20.0	87,178.1	86,435.0
Income quintile 3	18,212	20.0	65,828.7	65,773.8
Income quintile 2	18,212	20.0	46,723.2	47,243.1
Income quintile 1	18,212	20.0	21,081.8	20,642.6
Number of entitlements to special medicine reimbursement			0.5	0.0
Residents with entitlements to special medicine reimbursement	25,200	27.7		
Total number of residents	91,060			

Notes: Study population: non-student working-age (25–64) residents of Oulu in 2017–2018. Sex, age, education, and occupational class were measured at the beginning of 2017. Income and number of entitlements to special medicine were measured for the two-year period from 2017 to 2018.

**Table 2:** Descriptive statistics for medical and dentist visits during years 2017–2018.

	N	%	Mean	Median	IQR
Number of medical visits					
Total			13.8	8	15
Public			6.4	2	6
OHS			6.2	2	9
Private			1.2	0	1
Number of dentist visits			2.2	1	3
Residents with medical visits to					
Any sector	83,111	91.3			
Public	61,706	67.8			
OHS	50,523	55.5			
Private	36,025	39.6			
Residents with dentist visits	58,733	64.5			
Residents with					
Medical and dentist visits	55,250	60.7			
Only medical visits	27,861	30.6			
Only dentist visits	3483	3.8			
No medical or dentist visits	4466	4.9			

Note: Study population: non-student working-age (25–64) residents of Oulu in 2017–2018. Note that a resident may have medical visits in more than one sector. IQR stands for the interquartile range and is the difference between the 75th and 25th percentile.

## Data Availability

Due to legal restrictions and the data protection regulations of the administrative sources providing individual-level register data, the authors do not have the permission to make sensitive personal data available. For access to data on health services in the City of Oulu, Finnish Institute for Health and Welfare data, and data of the Social Insurance Institution of Finland, interested parties may apply to the centralized data permit authority Findata (https://www.findata.fi/en/, accessed on 1 December 2021), info@findata.fi. Applications for permission to access data on education and occupational class may be submitted to Statistics Finland (https://www.stat.fi/tup/mikroaineistot/index_en.html, accessed on 1 December 2021), tutkijapalvelut@stat.fi. Data on taxable income is available by application to the Finnish Tax Administration, verohallinto@vero.fi, P.O. Box 325, 00052 VERO.

## Supplementary Materials

The following are available online at https://www.mdpi.com/article/10.3390/ijerph182413337/s1. Table S1: Negative binomial model incidence rate ratios and 95% confidence intervals.

## Appendix A

**Table A1:** Logit odds ratios (OR) and 95% confidence intervals (CI) for having at least one dentist visit.

	Model 1			Model 2		
	OR	95% CI	*p*-Value	OR	95% CI	*p*-Value
Intercept	0.27	(0.25, 0.29)	<0.001	0.35	(0.33, 0.38)	<0.001
Total medical visits						
0 (ref.)	1					
1–2	1.43	(1.33, 1.53)	<0.001			
3–5	1.67	(1.58, 1.76)	<0.001			
6–10	1.98	(1.88, 2.10)	<0.001			
>10	2.6	(2.47, 2.75)	<0.001			
Public visits						
0 (ref.)				1		
1–2				1.17	(1.12, 1.22)	<0.001
3–5				1.31	(1.26, 1.36)	<0.001
6–10				1.55	(1.47, 1.64)	<0.001
>10				1.88	(1.78, 1.97)	<0.001
OHS visits						
0 (ref.)				1		
1–2				0.95	(0.89, 1.02)	0.176
3–5				1.03	(0.98, 1.07)	0.255
6–10				1.14	(1.09, 1.20)	<0.001
>10				1.39	(1.33, 1.45)	<0.001
Private visits						
0 (ref.)				1		
1–2				1.35	(1.30, 1.41)	<0.001
3–5				1.58	(1.52, 1.65)	<0.001
6–10				1.79	(1.63, 1.96)	<0.001
>10				2.36	(2.00, 2.80)	<0.001
Education						
Upper tertiary	1.26	(1.18, 1.34)	<0.001	1.23	(1.15, 1.31)	<0.001
Lower tertiary	1.38	(1.31, 1.46)	<0.001	1.35	(1.28, 1.43)	<0.001
Secondary	1.22	(1.16, 1.28)	<0.001	1.21	(1.15, 1.27)	<0.001
Basic (ref.)	1			1		
Occupational class						
U. non-manual employee	1.02	(0.97, 1.08)	0.455	1	(0.95, 1.06)	0.966
L. non-manual employee	1.02	(0.98, 1.07)	0.307	1.02	(0.97, 1.07)	0.507
Manual worker (ref.)	1			1		
Entrepreneur	1.25	(1.17, 1.34)	<0.001	1.08	(1.00, 1.16)	0.043
Other	1.11	(1.06, 1.17)	<0.001	1.06	(1.01, 1.12)	0.024
Income						
Quantile 5	2.33	(2.19, 2.47)	<0.001	2.46	(2.31, 2.62)	<0.001
Quantile 4	1.78	(1.69, 1.88)	<0.001	1.88	(1.77, 1.99)	<0.001
Quantile 3	1.59	(1.51, 1.67)	<0.001	1.64	(1.55, 1.73)	<0.001
Quantile 2	1.36	(1.30, 1.43)	<0.001	1.36	(1.30, 1.43)	<0.001
Quantile 1 (ref.)	1			1		
Sex						
Male (ref.)	1			1		
Female	1.59	(1.54, 1.65)	<0.001	1.49	(1.45, 1.54)	<0.001
Number of entitlements to special medicine reimbursements	1.01	(1.00, 1.03)	0.106	1	(0.99, 1.02)	0.581
Age group						
25–34 (ref.)	1			1		
35–44	1.13	(1.09, 1.17)	<0.001	1.13	(1.08, 1.17)	<0.001
45–54	1.64	(1.57, 1.70)	<0.001	1.62	(1.56, 1.69)	<0.001
55–64	2.03	(1.94, 2.12)	<0.001	1.96	(1.87, 2.05)	<0.001
