# Supplementary material for: Associations of Medical Visits with Dentist Visits: A Register-Linkage Study of a Working-Age Population in Finland
Source: Int J Environ Res Public Health. 2021 Dec 18;18(24):13337. doi: 10.3390/ijerph182413337 (PMC8703975; doi:10.3390/ijerph182413337)
Supplement: Supplementary file 1 [file ijerph-18-13337-s001.zip › ijerph-1478957-supplementary.pdf]

Table S1. Negative binomial model incidence rate ratios (IRR) and 95% confidence intervals (CI) for dentist visits.

	Model 1			Model 2		
	IRR	95% CI	P-value	IRR	95% CI	P-value
Intercept	0.83	(0.79, 0.87)	< 0.001	0.96	(0.92, 1.00)	0.076
Total medical visits						
0 (ref.)	1					
1-2	1.24	(1.18, 1.30)	< 0.001			
3-5	1.35	(1.31, 1.40)	< 0.001			
6-10	1.48	(1.43, 1.54)	< 0.001			
>10	1.78	(1.72, 1.84)	< 0.001			
Public visits						
0 (ref.)				1		
1-2				1.10	(1.07, 1.13)	< 0.001
3-5				1.18	(1.15, 1.21)	< 0.001
6-10				1.32	(1.28, 1.36)	< 0.001
>10				1.57	(1.52, 1.61)	< 0.001
OHS visits						
0 (ref.)				1		
1-2				0.96	(0.92, 1.00)	0.049
3-5				1.01	(0.98, 1.04)	0.445
6-10				1.05	(1.02, 1.08)	< 0.01
>10				1.16	(1.13, 1.19)	< 0.001
Private visits						
0 (ref.)				1		
1-2				1.11	(1.09, 1.14)	< 0.001
3-5				1.18	(1.15, 1.20)	< 0.001
6-10				1.30	(1.25, 1.37)	< 0.001
>10				1.50	(1.39, 1.62)	< 0.001
Education						
Upper tertiary	0.99	(0.96, 1.03)	0.729	0.99	(0.95, 1.03)	0.530
Lower tertiary	1.09	(1.05, 1.12)	< 0.001	1.08	(1.05, 1.12)	< 0.001
Secondary	1.09	(1.05, 1.12)	< 0.001	1.09	(1.06, 1.12)	< 0.001
Basic (ref.)	1			1		
Occupational class						
U. non-manual employee	0.96	(0.93, 0.99)	0.022	0.95	(0.92, 0.98)	< 0.01
L. non-manual employee	0.94	(0.92, 0.97)	< 0.001	0.94	(0.92, 0.97)	< 0.001
Manual worker (ref.)	1			1		
Entrepreneur	1.10	(1.06, 1.15)	< 0.001	1.02	(0.98, 1.06)	0.352
Other	1.06	(1.03, 1.09)	< 0.001	1.02	(0.99, 1.05)	0.249
Income						

	Model 1			Model 2		
	**IRR**	**95% CI**	**P-value**	**IRR**	**95% CI**	**P-value**
Quantile 5	1.35	(1.31, 1.40)	< 0.001	1.44	(1.39, 1.50)	< 0.001
Quantile 4	1.21	(1.18, 1.25)	< 0.001	1.29	(1.25, 1.33)	< 0.001
Quantile 3	1.17	(1.14, 1.21)	< 0.001	1.22	(1.18, 1.26)	< 0.001
Quantile 2	1.10	(1.07, 1.13)	< 0.001	1.11	(1.08, 1.15)	< 0.001
Quantile 1 (ref.)	1			1		
Sex						
Male (ref.)	1			1		
Female	1.23	(1.20, 1.25)	< 0.001	1.19	(1.17, 1.21)	< 0.001
Number of entitlements to special medicine reimbursement	1.04	(1.03, 1.05)	< 0.001	1.02	(1.02, 1.03)	< 0.001
Age group						
25-34 (ref.)	1			1		
35-44	1.11	(1.08, 1.13)	< 0.001	1.11	(1.08, 1.14)	< 0.001
45-54	1.41	(1.38, 1.44)	< 0.001	1.42	(1.38, 1.45)	< 0.001
55-64	1.58	(1.54, 1.62)	< 0.001	1.57	(1.53, 1.61)	< 0.001

Note: Study population: non-student working-age (25–64) residents of Oulu in 2017–2018. The outcome is the number of dentist visits. Model 1 and Model 2 are estimated from separate regressions. Model 1 corresponds to a model that uses the total number of medical visits. Model 2 separates medical visits to their respective health care sectors.
